# A remarkable Cornish site for ground-nesting bees and wasps

**DOI:** 10.3897/BDJ.12.e138021

**Published:** 2024-11-26

**Authors:** James B Whitfield, Sydney A Cameron

**Affiliations:** 1 University of Illinois Urbana-Champaign, Urbana, United States of America University of Illinois Urbana-Champaign Urbana United States of America; 2 Treveth, Lamorna, Penzance, United Kingdom Treveth Lamorna, Penzance United Kingdom

**Keywords:** Andrenidae, Apidae, Astatidae, Bembicidae, Chrysididae, Crabronidae, Halictidae, Megachilidae, Philanthidae, Pompilidae, Sphecidae, Hard Cliff, Maritime Heath

## Abstract

The south coast of the UK features a number of habitats rich in ground-nesting aculeate wasps and bees. Many of these are in predominantly sandy areas due to nesting requirements, but adjacent heathland may be especially critical in providing rich flower sources for these insects.

A surprisingly small transition zone between Hard Cliff and Maritime Heath habitats was found to support an unusually rich local fauna of ground-nesting bees and wasps, near the top of a promontory known as Carn Du, SE of Lamorna Cove in south-western Cornwall. In an area of partly exposed sandy soil measuring approximately 20 m^2^, more than twenty species (ten solitary bees, 10 aculeate wasps) were found during summer 2024, along with a handful of rarely observed species. We report the species found nesting there and illustrate many of them via field photographs.

## Introduction

Coastal areas in the United Kingdom are known to contain a variety of rich nesting sites for ground nesting solitary bees and wasps (Yeo and Corbet 1995, Falk and Lewington 2015, Benton 2017), especially Soft Cliff and Sand Dune habitats (Lake et al. 2020). Hard Cliff and Maritime Heath (Lake et al. 2020) are rich in flowers useful to these bees and wasps and often supply more limited areas of sandy or open soil for nesting sites of many species.

On 14 September 2023, we noted a site at Carn Du along the South West Coast Path (SWCP) in West Penwith, Cornwall, in the open transition zone between Hard Cliff and Maritime Heath with abundant nesting of the Bee Wolf *Philanthustriangulum* (Fabricius), as well as a few open burrows of several other (then unidentified) bee and wasp species. Bee and wasp activities were easily visible from the heavily used SWCP, but perhaps because of the location, which offers spectacular views over Lamorna Cove and the English Channel stretching NE toward Mounts Bay, few walkers appeared to notice the activity.

In 2024, a cool, wet spring (wettest since 1986 according to the Met Office (www.metoffice.gov.uk)) led to somewhat later than usual peak activity of many insects, including butterflies. We returned to the site several times in spring in 2024 and first noticed ground-nesting activity on 10 July, as Bee Wolves and the Green Eyed Flower Bee *Anthophorabimaculata* (Panzer) were digging nest burrows. Four days later, we spent several hours at the site and recorded a much higher diversity of at least 10 species using photography and net collection into small tubes for live examination. Thereafter, we returned to the site multiple times during the remainder of July and into early August to photograph and record the bees and wasps found nesting there. By the second week of August, most of the remaining activity was limited to *Philanthus*, the common *Tachysphex* and small *Lasioglossum* bees.

## Material and methods

We visited the site six times over 30 days for a total of approximately 10 hours of direct observations between 11:00 h and 16:50 h (depending on dry sunny periods). By 1700 h, activity declined significantly. We recorded GPS location with an iPhone 12 Pro. We used iPhones (11 and 12 Pro) and a Canon Powershot Elph 190 IS camera to photograph the nesting insects and nest openings. We marked six burrows temporarily with coloured pushpins to track bees entering and leaving nests. We netted smaller bees and wasps onsite and examined them in clear plastic tubes for photography and identification before releasing. A few specimens of these smaller wasps and bees required microscopic examination for identification; these were collected into 95% ethanol and later pinned and labelled.

### The aggregation site

**Figure 1.**
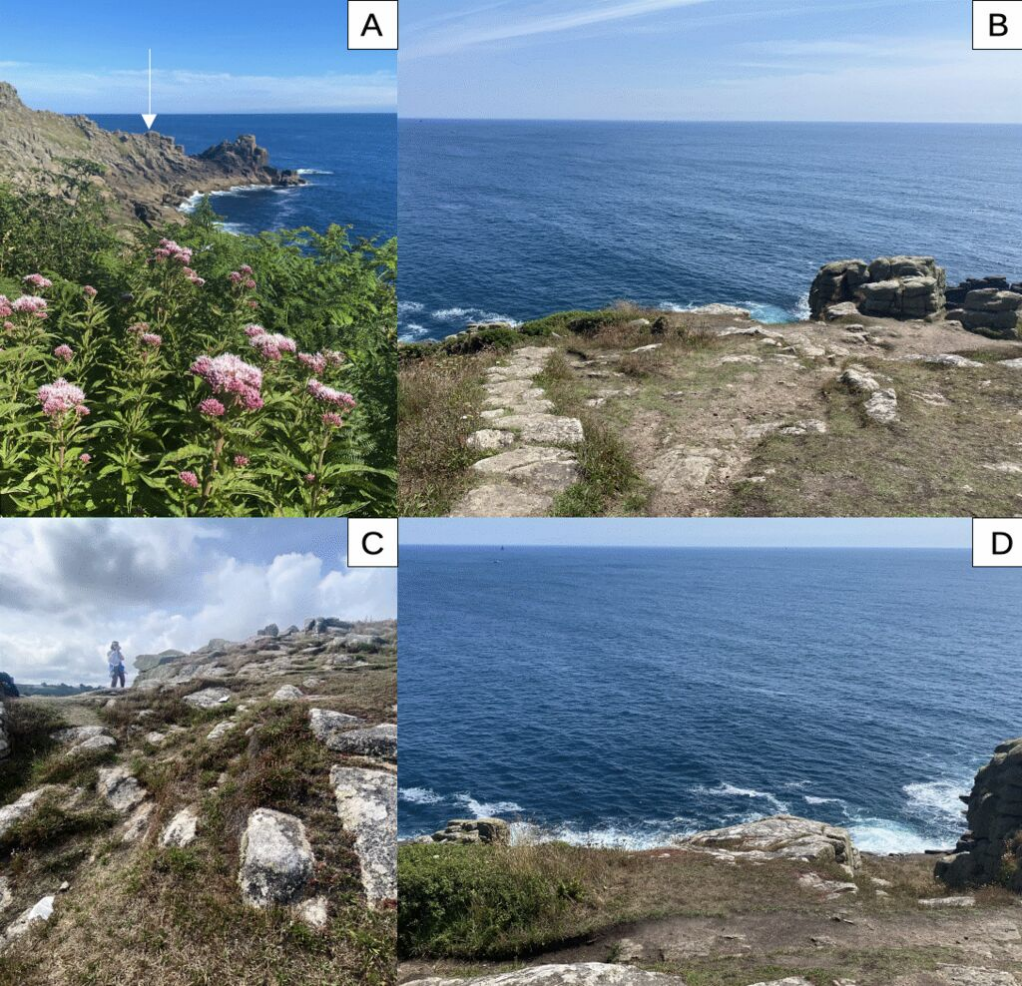
The aggregation site at Carn Du. **A** View to eastwards from across Lamorna Cove, with arrow indicating location of the aggregation site; **B** View from uphill of the site, showing larger open area and steps of the SWCP; **C** View of the site from below showing rocky edge of site; **D** Closer view of centre of **B**, showing dense part of the aggregation area in foreground.

The site is along the SWCP between Lamorna Cove and Mousehole, roughly 0.7 km from Lamorna Cove, near the top of the rocky peninsula of Carn Du (Fig. 1, 50°3’39”N, 5°33’17”W, grid reference SW 45700 23892, 20-30 m elevation above sea level). The SWCP (Fig. 1) traverses the upper portion of the sparsely vegetated transition zone between Rocky Cliff and Maritime Heath vegetation. The aggregation of nesting burrows was densest within a 5.1 m × 3.9 m open area between several large boulders and the surrounding heath (Fig. 1D, foreground: also visible in upper right of Fig. 1C and centre of Fig. 1B). Additional nesting activity extended down SE and SW facing slopes towards the sea, wherever there was relatively open space with low vegetation. Plants blooming in the area when surveys were conducted included: *Achilleamillefolium* (Yarrow), *Anthyllisvulneraria* (Kidney Vetch), *Armeriamaritima* (Seaside Thrift), *Cirsiumvulgare* (Spear Thistle), *Daucuscarota* (maritime form of Wild Carrot), *Ericacinerea* (Bell Heather), *Eupatoriumcannabinum* (Hemp Agrimony), *Hypochaerisradicata* (Cat’s-ear), *Jasionemontana* (Sheep’s-bit Scabious), *Lonicerapericlymenum* (Honeysuckle), *Rubusfruticosus* agg. (Bramble), *Sileneuniflora* (Sea Campion), *Thymuspolytrichus* (Wild Thyme), *Ulexgallii* (Western Gorse) and *Urticadioica* (Stinging Nettle).

### Identification and voucher specimens

Bees were identified using the keys and additional biological and geographical information in Falk and Lewington 2015 and Benton 2017; common names follow Falk and Lewington (2015). Wasps were identified using Yeo and Corbet 1995 and Brock 2021; common names follow Brock (2021). Chrysidid wasps were further checked using the keys in Morgan (1984), pompilid wasps using the keys in Day (1988) and other wasps using the keys in Richards (1980). A few identifications were also checked with respect to several recent sources of taxonomic clarifications and distributional records: Bogusch and Straka 2012, Wisniowski 2015, Baldock and Hawkins 2016, Straka 2016.

Larger bees and wasps belonging to relatively easily identifiable species were identified from photographs taken at the site. In the case of several of the wasps, their prey items (honey bees, weevils, shield bugs) were also helpful in identification.

## Data resources

Thirteen of the recorded species are represented by voucher specimens available from the authors. All records of species from the site were reported to the Bees, Wasps and Ants Recording Scheme (BWARS).

## Results

**Figure 2.**
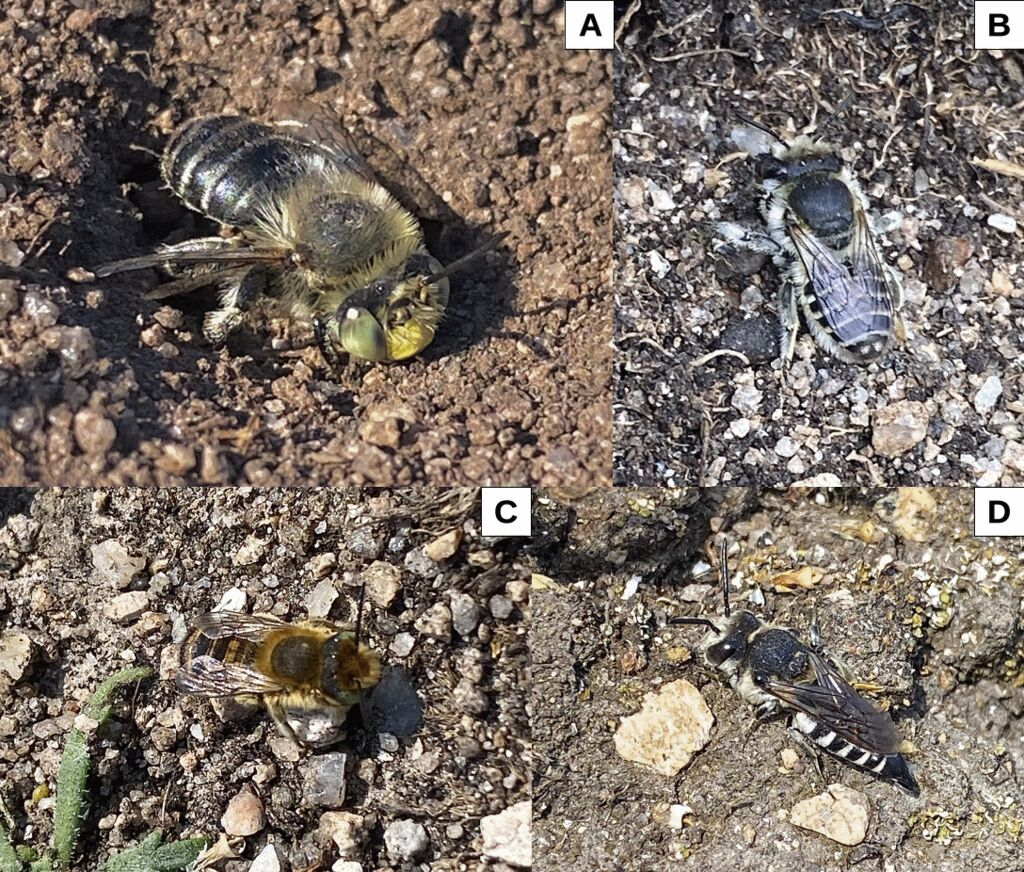
Selected bees from the aggregation site. **A** *Anthophorabimaculata* (Panzer) female at burrow entrance; **B** *Megachileleachella* Curtis female on ground near burrow; **C** *Megachilemaritima* (Kirby) on ground near burrow; **D** *Coelioxysconoideus* (Illiger) near host burrow.

**Figure 3.**
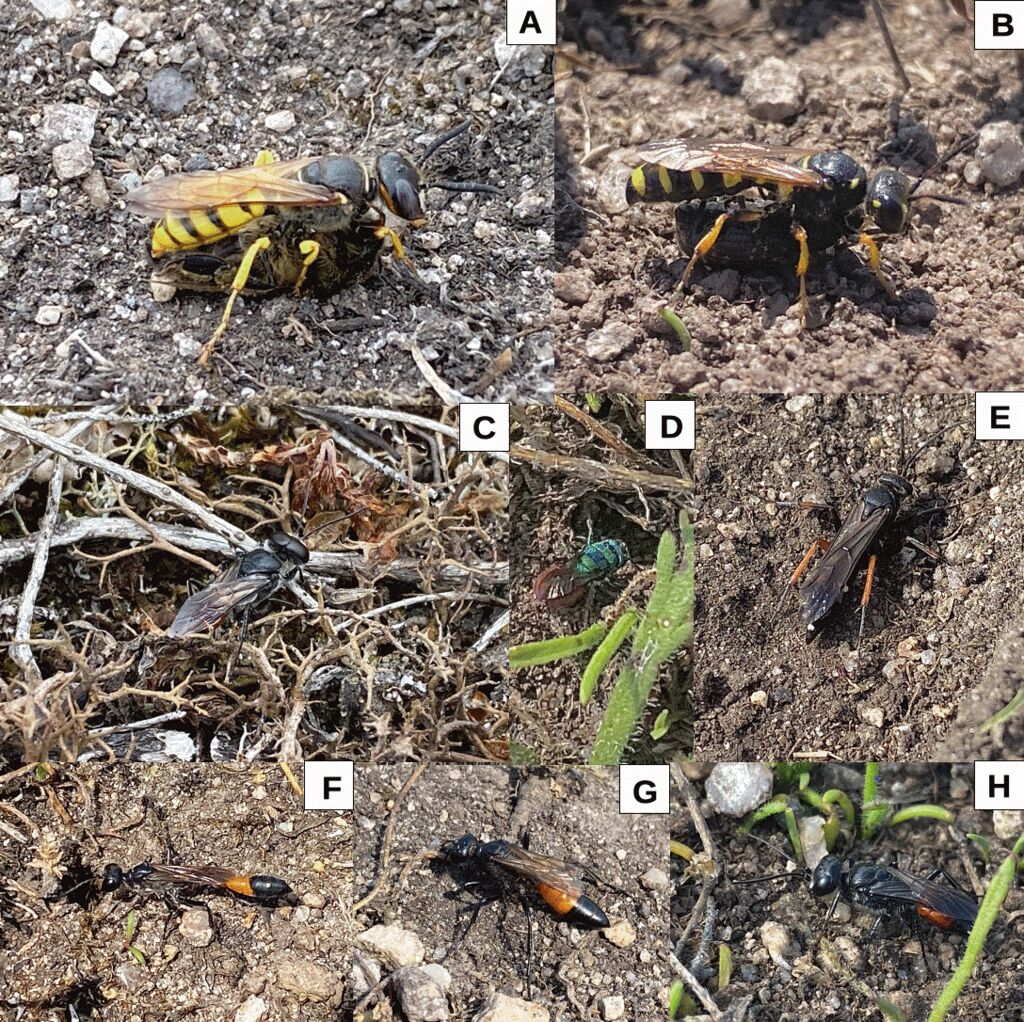
Selected wasps from the aggregation site. **A** *Philanthustriangulum* (Fabricius) female with prey honey bee carried beneath her; **B** *Cercerisarenaria* (Linnaeus) female with weevil prey slung underneath her; **C** *Astataboops* (Schrank) female on ground searching for prey items; **D** *Hedychridiumroseum* (Rossi) female investigating an *Astata* burrow; **E** *Episyronrufipes* (Linnaeus) female on ground near burrow; **F** *Ammophilasabulosa* (Linnaeus) female investigating burrow; **G** *Podaloniahirsuta* (Scopoli) female on ground at aggregation site; **H** *Tachysphexpompiliformis* (Panzer) female searching for grasshopper nymphs at site.

Table 1 lists the species we found active at the site and were able to identify. Ten species of bees, from eight genera in four families and ten species of wasps from ten genera in seven families, were identified from the site. Examples of the photographed bees and wasps are represented in Figs 2, 3.

A few additional small bees (principally small Halictidae) and wasps (principally Chrysididae) observed at the site were not identified due to our inability to photograph or collect them. These represent an additional four to five species, none of which appeared to be common at the site. In addition, several small flies (possibly *Miltogramma*) appeared to be hovering about some of the nest openings, but were not collected.

## Discussion

The species we found simultaneously nesting or parasitising nests at this site represent a surprisingly high diversity within a relatively small area, especially in a year known for low insect populations (attributed to poor early season weather) and also considering only the mid-late summer season. The absence of some host species for observed parasites and vice versa suggests that we could find a few additional species at the aggregation in the future.

## Conclusions

This site represents an example of extraordinarily high species richness of diverse aculeate Hymenoptera in an extremely small area and a prime example of the conservation value of Cornwall’s South West Coast Path. While some sandy habitats such as sand quarries (e.g. Twerd et al. (2021)) may have greater overall aculeate diversity, the spatially limited transition zone we studied between Hard Cliff and Maritime Heath habitats is invaluable for additional studies of nesting behaviour and species interactions. Continued protection of such sites is critical for the maintenance of insect biodiversity in a world of declining species richness.

## Figures and Tables

**Table 1.** Hymenoptera identified from the Carn Du aggregation.

**Family**	**Genus**	**Species**	**Common name**	**Notes**
**Bees**				
Andrenidae	*Andrena*	*pilipes* Fabricius	Black mining bee	few at the site
Andrenidae	*Panurgus*	*banksianus* (Kirby)	Large shaggy bee	few at the site
Apidae	*Anthophora*	*bimaculata* (Panzer)	Green-eyed flower bee	few at the site
Apidae	*Nomada*	*rufipes* Fabricius	Black-horned nomad bee	few at the site
Halictidae	*Lasioglossum*	*morio* (Fabricius)	Green furrow bee	few at the site
Halictidae	*Sphecodes*	*ephippius* (Linnaeus)	Bare-saddled blood bee	host: probably *Lasioglossum*
Halictidae	*Sphecodes*	*monilicornis* (Kirby)	Box-headed blood bee	host: probably *Lasioglossum*
Megachilidae	*Coelioxys*	*conoideus* (Illiger)	Large sharp-tail bee	host: *Megachilemaritima*
Megachilidae	*Megachile*	*leachella* Curtis	Silvery leafcutter bee	abundant at the site
Megachilidae	*Megachile*	*maritima* (Kirby)	Coast leafcutter bee	less common than *leachella* at site
**Wasps**				
Chrysididae	*Hedychridium*	*roseum* (Rossi)	Dull cuckoo wasp	hosts: *Astata*, *Tachysphex*
Chrysididae	*Hedychrum*	*nobile* Scopoli	Noble cuckoo wasp	host: *Cercerisarenaria*
Pompilidae	*Episyron*	*rufipes* (Linnaeus)	Red-legged spider wasp	prey: orb-web spiders
Astatidae	*Astata*	*boops* (Schrank)	Shieldbug digger wasp	prey: shield bug nymphs
Bembicidae	*Harpactus*	*tumidus* (Panzer)	White-spotted digger wasp	prey: cercopid bugs
Crabronidae	*Tachysphex*	*pompiliformis* (Panzer) aggregate	Common Tachysphex	prey: grasshopper nymphs
Philanthidae	*Cerceris*	*arenaria* (Linnaeus)	Sand-tailed digger wasp	prey: weevils
Philanthidae	*Philanthus*	*triangulum* (Fabricius)	European bee-wolf	prey: honey bees
Sphecidae	*Ammophila*	*sabulosa* (Linnaeus)	Red-banded sand wasp	prey: caterpillars
Sphecidae	*Podalonia*	*hirsuta* (Scopoli)	Hairy sand wasp	prey: caterpillars
